# Improving the Effectiveness of Intentional Rounding: A Quality Improvement Project

**DOI:** 10.1155/nrp/7573541

**Published:** 2026-03-13

**Authors:** Yingxin Peng, Shanqin Qiu, Dan Zhao, Jie Wang

**Affiliations:** ^1^ Department of Nursing, The Fourth Affiliated Hospital of Soochow University (Suzhou Dushu Lake Hospital), No. 9 Chongwen Road, Suzhou 215000, Jiangsu, China

**Keywords:** adverse events, intentional rounding, patient safety, quality

## Abstract

**Background:**

Ineffective intentional rounding (IR) may lead to missed care opportunities and an increased risk of adverse events. Quality improvement (QI) strategies may enhance nurses’ compliance with rounding protocols and improve patient safety.

**Objective:**

To evaluate the impact of a multifaceted QI program on the rate of effective IR and the incidence of nursing‐related adverse events.

**Methods:**

This pre–post study was conducted at a tertiary hospital. The department of nursing‐level interventions included the following: revising the scope of required hourly patient rounds to direct nurses’ attention toward elderly patients and those at high risk of falls; mandating that nurses could only document round completion by scanning either patient wristbands or bedside QR codes containing patient information; and providing standardized, organization‐wide training. Ward‐level interventions included disease‐specific training, high‐risk time reminders, establishing standardized effective round assessment forms, monthly reviews by liaison nurses, and regular random checks by the QI team. Effective round rates and adverse events (including unplanned extubation, intravenous injection safety events, and falls) were compared before and after implementation.

**Outcome:**

The timely rounding rate for first‐level nursing increased significantly from 77.46% in 2023 to 91.89% in Q4 2024 (*χ* ^2^ = 3561.443, *p* < 0.001). The proportion of unplanned extubations attributed to ineffective rounding decreased from 20.69% (6/29 events) to 0% (0/12 events), and similar attributions for intravenous safety incidents decreased from 61.54% (8/13) to 30.77% (4/13); however, these reductions were not statistically significant (*p* = 0.106 and *p* = 0.119, respectively). Process audit data indicated that gains in some areas were not fully sustained across all quarters.

**Conclusion:**

The multifaceted QI program improved nurses’ compliance with IR processes and was associated with improvements in selected nursing safety indicators. Sustaining these gains requires continuous monitoring, targeted feedback, and adaptive reinforcement strategies.

## 1. Introduction

Intentional rounding (IR) refers to a structured and proactive nursing practice involving purposeful, regular bedside visits aimed at assessing patients’ clinical status, addressing fundamental care needs, ensuring safety, and enhancing the quality of care [1, 2]. Previous studies have reported that IR is generally associated with improved patient perceptions of care, enhanced feelings of safety, reduced incidence of inpatient falls, and improved nurse satisfaction, suggesting its potential value as a patient‐centered nursing intervention [3–6]. Importantly, the underlying mechanism of IR is thought to lie in increased nurse–patient interaction and anticipatory care, rather than reactive task‐based nursing [2].

In China, inpatient nursing care is delivered within a nationally mandated tiered nursing system issued by the National Health Commission (NHC). Patients are classified into four nursing levels—special care, Level 1 care, Level 2 care, and Level 3 care—based on clinical condition and self‐care ability. Each level corresponds to a prescribed minimum rounding frequency, ranging from continuous close observation (special care) to hourly (Level 1), two‐hourly (Level 2), and three‐hourly (Level 3) IR [7]. Its main objective is to optimize the allocation of limited nursing resources by matching the intensity of care to the needs of patients, thereby standardizing nursing practices. In tertiary general hospitals, the majority of inpatients are typically assigned to Level 1 or Level 2 care, while only a small proportion receive special care, resulting in substantial variation in mandated rounding frequency across patient groups.

Despite the widespread adoption of tiered IR, evidence regarding its real‐world implementation quality and effectiveness remains limited. High nursing workloads, competing clinical demands, and an overemphasis on documentation rather than the content of rounding may undermine the intended benefits of IR, potentially leading to missed care opportunities and compromised patient safety [8–10].

Our hospital is a tertiary general hospital characterized by high patient turnover and sustained nursing workload pressure. In consideration of clinical workflow realities while preserving the goal of hourly observation, timely rounding for first‐level care was operationally defined as having an interval of ≤ 75 min between consecutive rounds. The compliance rate with this standard in 2023 was 77.46%. Furthermore, internal incident reporting identified several safety‐related adverse events temporally associated with lapses in IR. These observations not only show that the implementation of IR needs improvement but also highlight the importance of improving the quality of IR.

The “Plan–Do–Check–Act” (PDCA) process is a procedural, standardized, and scientific quality management method that has been widely used in nursing management and nursing practice [11, 12]. This quality improvement (QI) project used PDCA as a guiding tool, aimed to establish a standardized IR evaluation tool in our hospital, conduct it in the hospital, and improve the effectiveness of IR and ensure patient safety.

## 2. Methods

### 2.1. Information Collection

This study is divided into two phases: the first phase is before the PDCA intervention (January–March 2024), and the second phase is the improvement period (April 2024–December 2024). The project was conducted in a tertiary general hospital with approximately 800 inpatient beds. A total of 27 wards were included, covering a broad range of clinical specialties, including internal medicine, surgery, the emergency department, and the intensive care unit (ICU). Although the hospital does not have a dedicated geriatric ward, older adults are routinely admitted to medical and surgical wards as part of routine clinical practice. This study was conducted according to the guidelines of the Standards for Quality Improvement Reporting Excellence [13] (Supporting Table S1).

#### 2.1.1. Plan

A hospital‐wide QI team was established, comprising a team leader (the department head nurse), a deputy team leader, core team members, and ward‐level liaisons. Specifically, nurses from each of the hospital’s 27 wards (hereafter referred to as nursing units) served as liaisons responsible for coordinating QI activities and facilitating data collection. The overarching aim of the QI initiative was to improve the effectiveness of IR within the tertiary general hospital.

To assess IR effectiveness, a standardized evaluation form (Supporting Table S2) was developed in accordance with NHC’s requirements and the action plan for further improvement of nursing services (2023–2025). The form was designed to evaluate whether nurses conducted structured and purposeful rounds. The evaluation tool comprised 8 domains and 19 specific criteria, with a total possible score of 100 points. The 8 domains of the evaluation tool include nursing level, patient positioning, infusion and blood transfusion inspection priorities, monitor inspection priorities, pipeline inspection priorities, oxygen therapy inspection priorities, specialty‐specific inspection priorities, and risk group inspection priorities. Scores below 90 were classified as unsatisfactory.

In March 2024, a cross‐sectional on‐site audit was conducted at 3 time points (day shift: 11:00–12:00; evening shift: 18:00–19:00; and night shift: 06:00–07:00) in 7 wards, including 3 medical wards, 3 surgical wards, and 1 ICU. In each ward, 10 patients classified as requiring first‐level nursing care were randomly selected, yielding a total sample of 70 patients. For the purposes of this QI evaluation, compliance was assessed primarily at the ward (the nursing unit) level rather than the individual patient level. A ward was classified as noncompliant if at least 1 evaluated patient failed to meet the predefined IR standards. This conservative approach was adopted to identify wards in which IR processes were not consistently and reliably implemented, reflecting a “minimum standard” perspective commonly used in quality management to flag systemic weaknesses rather than individual performance variation.

Only two of the seven wards met the compliance standard. Analysis based on the 8 domains of the evaluation checklist indicated that the most prominent deficiencies at the domain level were related to monitor inspection priorities, accuracy of nursing level classification, and pipeline inspection priorities. Further examination of the specific evaluation criteria revealed that the five most frequently observed problems were as follows: (1) inconsistency between the assigned nursing level and the patient’s actual clinical condition or failure to conduct nursing rounds at the prescribed frequency; (2) inappropriate settings of monitoring device alarms; (3) inadequate implementation of preventive measures for high‐risk patients, including those at risk of falls, pressure injuries, and deep vein thrombosis; (4) insufficient awareness of key points of specialty observation and potential complications; and (5) poor adherence to standards for catheter care, including cleaning, maintenance of patency, and secure fixation.

Furthermore, a retrospective analysis of adverse events in 2023 revealed that ineffective or delayed IR was a major contributing factor to intravenous safety incidents, patient falls, and unplanned extubations.

**FIGURE 1:**
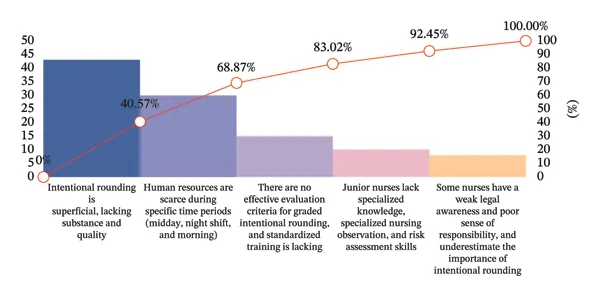
Pareto analysis of factors contributing to ineffective intentional rounding.

During the baseline assessment phase, we conducted a Pareto analysis (Figure 1) to identify and prioritize the factors contributing to ineffective purposeful rounding. The bar graph represents the frequency with which each factor was identified in the wards during on‐site audits, while the cumulative curve represents the cumulative percentage of the total number of identified problems.

#### 2.1.2. Do

The nursing department has revised the tiered nursing care system. According to the nursing tiered standards, patients with stable conditions who are critically ill, those with unstable conditions or conditions that may change at any time, those requiring strict bed rest after surgery or during treatment, and those with severe dependence on self‐care abilities are classified as Level 1 care patients. To raise nurses’ awareness of elderly patients and those at risk of falls, patients aged ≥ 75 years or ≥ 65 years with serious chronic diseases are classified as Level 1 care patients. In addition, patients with severe osteoporosis or a history of falls within the past 3 months are also classified as Level 1 care patients. These patients are considered to be at higher risk of clinical deterioration or safety‐related events, thus requiring more intensive and targeted nurse–patient interaction.

The nursing department has implemented closed‐loop information management. Each inpatient has a unique QR code, which nurses can scan to view the patient’s basic information and complete rounds. We have closed other methods that could generate QR codes for all patients in the ward at once; nurses can only complete IR by scanning the patient’s wristband or bedside QR code.

The nursing department organized training for all nurses on nursing assessment tools, comprehensive skills, and catheter care. Three training sessions on nursing assessment tools were conducted to enable nurses to correctly apply commonly used nursing risk assessment tools, such as the Braden scale, fall risk assessment tools, and unplanned extubation risk assessment tools, thereby accurately identifying high‐risk patients and implementing appropriate preventative measures in a planned manner. Comprehensive patient assessment training emphasized holistic patient evaluation to support early detection of nursing‐related problems during routine care and planned rounds. In addition, specialized training on the examination and fixation of indwelling devices was provided. Although nurses typically receive training on device placement, this training focused on continuous assessment and fixation during rounds, rather than initial insertion techniques. The training covered common types of indwelling devices used in the ward, including endotracheal tubes, drainage tubes, urinary catheters, and vascular access devices.

In addition, the QI team conducted a dedicated training session for all QI team members on the standards for effective IR. Departmental liaisons subsequently provided training on these standards to all nurses within their respective wards, ensuring consistent understanding and application of effective rounding requirements.

At the ward level, a visualized patient information panel at the nursing station displayed real‐time information on critically ill patients and patients at high risk of falls, pressure injuries, and unplanned extubation. Head nurses and clinical instructors used this information to focus their supervision on high‐risk patients and critical time periods, monitoring the implementation of IR. An automated rounding reminder function was embedded in the ward nursing information system, which is part of the hospital’s electronic nursing documentation platform used for task management and nursing record entry. During predefined high‐risk time periods (e.g., midday and early morning), the system automatically generated reminders to prompt nurses to complete planned rounds, thereby reducing missed or delayed rounding.

During the Check phase, liaison nurses in each ward conducted monthly assessments using the standardized evaluation form for effective IR. For each ward, 10 patients receiving Level 1 care were selected based on predefined criteria. To reduce selection bias and ensure representativeness, the sample included five patients cared for by senior bedside nurses (N2–N3) and five patients cared for by junior bedside nurses (N0–N1).

Eligible patients were those classified as Level 1 care at the time of assessment and present in the ward during the designated evaluation period. Patients were selected from the current ward census rather than being chosen based on clinical outcomes or perceived performance.

In June and September, the QI team conducted independent random inspections of IR. These inspections covered Level 1 care patients across day, evening, and night shifts to capture potential variability related to staffing patterns and workload at different times.

#### 2.1.3. Action

In the Action phase, the QI team systematically summarized and analyzed the problems identified during each PDCA cycle. On a quarterly basis, the QI team conducted a comprehensive aggregation and analysis of the issues that emerged within the corresponding quarter, including deficiencies in hierarchical IR implementation and deviations from standardized procedures.

Based on the results of this quarterly analysis, targeted adjustment strategies were discussed and formulated in a timely manner, such as refining training content, optimizing reminder mechanisms, and strengthening supervision for high‐risk patients and critical time periods. These adjustments were subsequently incorporated into the planning and implementation of the next PDCA cycle, thereby facilitating iterative refinement of the intervention process.

### 2.2. Observation Indicators

After implementing the QI interventions for effective IR, we compared the incidence of adverse events related to ineffective rounds, the rate of timely IR, and the number of problems identified through the standardized evaluation form between 2023 and 2024.

### 2.3. Data Collection

Nurses conducted IR by scanning the QR codes on patients’ wristbands or bedside cards using PDAs. The information technology department extracted the exact timing of IR and corresponding patient‐level information from the electronic nursing documentation system.

Given the large volume of IR records generated across all wards, a predefined sampling strategy was adopted to enable standardized and manageable data analysis. Specifically, IR records from the first five consecutive days of each month were extracted from seven representative wards, including 3 medical wards, 3 surgical wards, and 1 ICU. These wards were selected to reflect different clinical care contexts and nursing workloads.

All nursing levels have defined rounding requirements; however, only patients receiving special or first‐level nursing care were included, as these groups require frequent rounds and are therefore most appropriate for evaluating rounding timeliness. An IR was considered timely if the interval between two consecutive rounds did not exceed 75 min, allowing a reasonable operational tolerance for an hourly rounding requirement in real‐world clinical practice.

### 2.4. Data Analysis

Statistical analysis was performed using SPSS 26.0. Enumeration data were expressed as frequencies and percentages, and intergroup comparisons were performed using the chi‐square test or Fisher’s exact test. A *p* value of less than 0.05 was considered statistically significant.

## 3. Results

### 3.1. Comparison of Adverse Events

In 2023, there were 29 unplanned extubations, 13 venous safety incidents, and 21 falls. In 2024, there were 12 unplanned extubations, 13 venous safety incidents, and 22 falls. The proportion of venous safety incidents and unplanned extubations due to ineffective patrols decreased, but the difference was not significant (Table 1).

**TABLE 1:** Comparison of unplanned extubation before and after intervention.

	Unplanned extubation events due to ineffective patrol (%)	Venous safety incidents caused by ineffective patrol (%)
Before intervention (2023)	6 (20.69%)	8 (61.54%)
After intervention (2024)	0 (0)	4 (30.77%)
*p*	0.106	0.119

### 3.2. Special and First‐Level Nursing Inspections

In the first quarter of 2024, a total of 88,037 inspections were recorded across the seven wards, 10,667 of which were not timely, for a timely inspection rate of 87.88%. In the fourth quarter of 2024, a total of 96,147 inspections were recorded across the seven wards, 7797 of which were not timely, for a timely inspection rate of 91.98%. Table 2 presents the inspection data of seven wards in four quarters in 2024. The difference between the two figures was significant (chi‐square value = 3561.443, *p* < 0.001).

**TABLE 2:** Inspection data of seven wards in four‐quarters in 2024.

Period	Number of records	Number of inspections not recorded in time	Proportion of timely inspections (%)
First quarter	88,037	10,667	87.88
Second quarter	89,243	8986	89.93
Third quarter	97,878	9104	90.70
Fourth quarter	96,147	7797	91.89

### 3.3. Inspection Results

**FIGURE 2:**
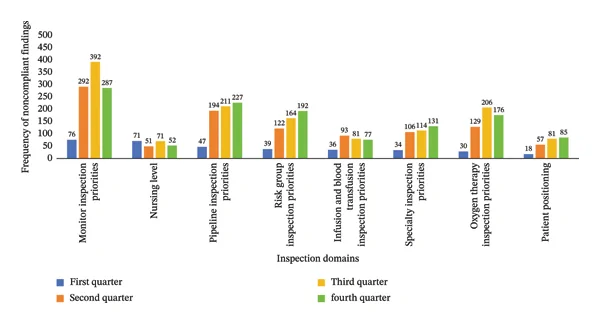
Quarterly trends in the frequency of noncompliant findings across major planned rounding inspection domains.

In the first quarter of 2024, only 2 of the 7 nursing units inspected met the compliance standards for effective planned rounds. Following the implementation of continuous QI measures, all units successfully passed subsequent monthly inspections. Figure 2 shows the distribution of quarterly noncompliance findings across key inspection areas.

**FIGURE 3:**
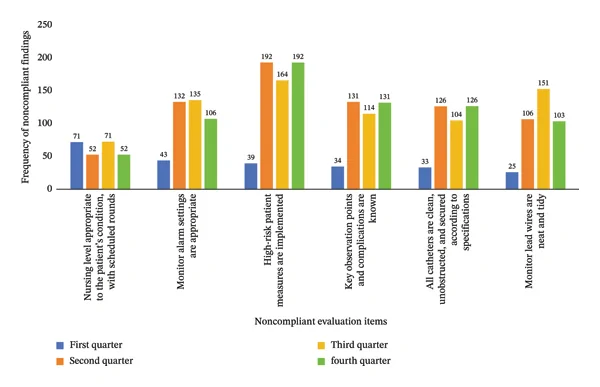
Quarterly comparison of the six most frequent noncompliant items among planned rounding evaluation criteria.

We compared the six most frequent issues found among the 19 criteria in the first quarter. Figure 3 shows the frequency of these six issues in each quarterly inspection. Higher values indicate more frequent deviations from planned rounding standards.

## 4. Discussion

This QI implemented a multifaceted intervention to enhance the effectiveness of IR within a tiered nursing care framework. The primary finding was that although the intervention significantly improved the timeliness and compliance of IR, these process improvements did not translate into a statistically significant reduction in related adverse events, including unplanned extubation and intravenous therapy–related safety events. In addition, audit data indicated that improvements in certain process indicators were not fully sustained throughout the observation period.

IR is an indispensable component of daily nursing practice. The multifaceted strategies implemented at both the hospital and ward levels in this study effectively increased nurses’ awareness of and adherence to structured rounding, thereby improving the timeliness of IR. This finding is consistent with previous studies demonstrating that purposeful and timely rounding constitutes a best‐practice intervention. The introduction of a standardized evaluation form to assess rounding effectiveness, together with monthly self‐audits and periodic random inspections, further embedded these practices into routine workflows. Existing evidence suggests that reinforcing structured rounding protocols through education, technological support, and managerial oversight can significantly enhance nurses’ compliance and situational awareness, thereby strengthening patient monitoring and facilitating early problem detection [14].

Structured rounding programs have been shown to anticipate patient needs by increasing monitoring frequency, enhancing situational awareness, and promoting proactive nursing care, which may contribute to reductions in adverse outcomes [15, 16]. However, our findings indicate that improvements in IR alone were insufficient to produce a significant reduction in adverse events associated with ineffective or delayed rounding. Several potential explanations may account for this result. First, outcomes such as patient falls are influenced by multiple factors, including underlying medical conditions, treatments, psychological and cognitive status, and environmental conditions [17, 18]; therefore, reliance on fall risk identification and rounding alone may be inadequate to achieve measurable improvements in fall‐related outcomes. Second, the study design and sample size were not powered to detect changes in relatively low‐frequency adverse outcomes such as unplanned extubation and intravenous safety events, suggesting that observed process improvements may not have been sufficient to overcome these broader determinants of patient harm. Finally, previous studies reporting reductions in adverse events following the implementation of IR have shown substantial heterogeneity in study design, intervention components, and outcome definitions, and high‐quality evidence establishing a direct causal relationship remains limited [19, 20].

Analysis of quarterly audit data further revealed that although the frequency of noncompliance decreased after the initial intervention phase, a partial rebound of certain indicators occurred in subsequent quarters. This nonlinear pattern of improvement is common in continuous QI initiatives, particularly those that rely primarily on behavioral change rather than structural redesign [21]. Competing clinical priorities, gradual declines in staff vigilance, and limited reinforcement mechanisms may contribute to the erosion of early gains. These findings underscore the importance of ongoing monitoring, timely feedback, and adaptive intervention strategies to sustain process improvements over time.

Overall, this study highlights both the potential and the limitations of rounding‐focused QI efforts. While multifaceted interventions can enhance the consistency and timeliness of nursing rounds, translating these process improvements into measurable and sustained patient safety outcomes remains challenging. Future initiatives may benefit from longer follow‐up periods, broader sampling strategies, and the integration of task‐specific tools to strengthen the linkage between rounding processes and clinical outcomes.

## 5. Limitations

Several limitations should be acknowledged in this study. First, the project was conducted in a single tertiary hospital, which may limit the generalizability of the findings to other healthcare settings with different patient populations, staffing models, or organizational cultures. Second, although the implementation included standardized evaluation criteria and periodic inspections, data collection relied partly on manual audits, which may have been subject to observer bias or inconsistency among quality‐control personnel. Finally, while improvements were sustained for some indicators, certain first‐level problems showed recurrence in later quarters, suggesting that longer follow‐up and adaptive reinforcement strategies are needed to evaluate long‐term effectiveness.

## 6. Conclusion

This QI initiative demonstrated that a multifaceted, multilevel intervention can effectively enhance nurses’ adherence to IR and significantly improve the timeliness of rounds within a graded nursing care framework. While improvements in rounding performance were achieved, corresponding reductions in nursing‐related adverse events were not consistently observed, underscoring the complexity of translating process improvements into sustained patient‐safety outcomes.

The observed fluctuations in selected process indicators over time further suggest that gains achieved through behavior‐focused interventions may attenuate without continuous reinforcement. These findings emphasize that IR should be viewed not as a one‐time intervention but as an ongoing organizational practice requiring sustained monitoring, structured feedback, and adaptive quality control strategies.

## Funding

The authors have not declared a specific grant for this research from any funding agency in the public, commercial or not‐for‐ profit sectors.

## Consent

The authors have nothing to report.

## Conflicts of Interest

The authors declare no conflicts of interest.

## Supporting Information

Additional supporting information can be found online in the Supporting Information section.

## Supporting information


**Supporting Information 1** Supporting Table S1: Revised Standards for Quality Improvement Reporting Excellence (SQUIRE 2.0). Table S1 is a standardized evaluation form for the effectiveness of graded nursing care implemented by the researcher’s hospital, which includes eight evaluation items: nursing level, patient positioning, infusion and blood transfusion inspection points, monitor inspection points, pipeline inspection points, oxygen therapy inspection points, specialist inspection points, and risk group inspection points.

**Supporting Information 2** Supporting Table S2: The standardized evaluation form for effective hierarchical IR.

## Data Availability

The data that support the findings of this study are available upon request from the corresponding author. The data are not publicly available due to privacy or ethical restrictions.
