# Supplementary material for: Improving the Effectiveness of Intentional Rounding: A Quality Improvement Project
Source: Nurs Res Pract. 2026 Mar 13;2026:7573541. doi: 10.1155/nrp/7573541 (PMC13459643; doi:10.1155/nrp/7573541)
Supplement: Supplementary file 1 [file NRP-2026-7573541-s001.docx]

**The standardized evaluation form for effective hierarchical intentional rounding**

| **First-level index inspections** | **Secondary indicator inspection** | **Deduction criteria** |
| --- | --- | --- |
| 1.Nursing level (15 points) | 1.1Nursing level appropriate to the patient's condition, with scheduled rounds | - No graded nursing order was prescribed – 3 pts - The assigned nursing level does not match the patient’s condition –3 pts - Failure to conduct rounding according to the assigned nursing level and schedule, or missed rounding; each non-compliance –3 pts |
| 2.Patient positioning (5 points) | 2.1Position appropriate to patient’s condition | - Patient’s position inconsistent with condition –5 pts |
| 3. Infusion and blood transfusion inspection priorities (15 points) | 3.1 Infusion/transfusion rate meets prescription | - Rate inconsistent with condition or prescription –5 pts - Incorrect vascular access device selection –5 pts - Redness/swelling/extravasation at puncture site –5 pts |
|  | 3.2 Correct vascular access device selected |  |
|  | 3.3 No redness, swelling, or extravasation at puncture site; secure fixation |  |
| 4. Monitor inspection priorities (15 point) | 4.1 Alarm settings appropriate | - Alarm settings unreasonable –2 pts - Leads detached –3 pts - Monitoring interval unreasonable –3 pts - Ineffective monitoring (failure to monitor timely) –5 pts |
|  | 4.2 Leads connected clearly and correctly |  |
|  | 4.3 Leads connected clearly and correctly |  |
| 5.Pipeline inspection priorities (20 points) | 5.1 Catheters labeled, unobstructed, fixed appropriately | - Catheter obstructed, contaminated, or dislodged –1 pt - Labeling non-standard/incomplete/inaccurate –2 pts - Inadequate catheter fixation –2 pts - Labels unclear, damaged, or incorrect –2 pts - Catheter indwelling time exceeds limit –2 pts - Untimely response to abnormal drainage –2 pts - Failure to record abnormalities –2 pts - Replacement missed or incorrect –2 pts - Drainage bag not emptied –2 pts - Inaccurate drainage records –2 pts |
|  | 5.2 Catheter labels complete and accurate |  |
|  | 5.3 Catheters replaced as required |  |
|  | 5.4Drainage volume/characteristics abnormal → reported and recorded |  |
|  | 5.5 Drainage fluid/dressings replaced as required with aseptic technique |  |
|  | 5.6 Drainage records accurate |  |
| 6.Oxygen therapy inspection priorities (10 points) | 6.1Oxygen therapy process consistent with condition & records | - Flow inconsistent with condition –2 pts - Recorded vs. actual flow mismatch –2 pts - Nasal cannula/mask displaced or improper –2 pts - Patient complains of dyspnea/frequent respiration –2 pts - Unreasonable device replacement –2 pts |
|  | 6.2 Oxygen signage/device appropriate |  |
|  | 6.3 Documentation accurate, timely, complete; device meets requirements |  |
| 7. Specialty inspection priorities (10 points) | 7.1Nurse aware of specialty-specific requirements and complications | - Lack of knowledge of specialty nursing requirements –2 pts - Lack of awareness of complications –2 pts |
| 8.Risk group inspection priorities (10 points) | 8.1Implementation of safety measures for high-risk patients | - Missing one preventive measure for high-risk patient (falls, VTE, pressure injury) –3 pts - Missing side-rail precautions for patients with delirium, agitation, cognitive impairment, or dementia –3 pts - No companion for high-risk patient –2 pts |
