# Supplementary material for: Improving the Effectiveness of Intentional Rounding: A Quality Improvement Project
Source: Nurs Res Pract. 2026 Mar 13;2026:7573541. doi: 10.1155/nrp/7573541 (PMC13459643; doi:10.1155/nrp/7573541)
Supplement: Supplementary file 2 [file NRP-2026-7573541-s002.docx]

**Revised Standards for Quality Improvement Reporting Excellence (SQUIRE 2.0) September 15, 2015**

| **Text Section and Item**  **Name** | **Section or Item Description** | **page** |
| --- | --- | --- |
| **Title and Abstract** |  |  |
| **1. Title** | Indicate that the manuscript concerns an initiative to improve healthcare (broadly defined to include the quality, safety, effectiveness, patient- centeredness, timeliness, cost, efficiency, and equity of healthcare) | 2 |
| **2. Abstract** | a. Provide adequate information to aid in searching and indexing b. Summarize all key information from various sections of the text using the abstract format of the intended publication or a structured summary such as: background, local problem, methods, interventions,   results, conclusions | 2-3 |
| **Introduction** | *Why did you start?* |  |
| **3. Problem** **Description** | Nature and significance of the local problem | 3 |
| **4. Available knowledge** | Summary of what is currently known about the problem, including relevant previous studies | 3 |

| **5.** **Rationale** | Informal or formal frameworks, models, concepts, and/or theories used to explain the problem, any reasons or assumptions that were used to develop the intervention(s), and reasons why the intervention(s) was expected to work | 3-4 |
| --- | --- | --- |
| **6. Specific aims** | Purpose of the project and of this report | 4 |
| **Methods** | *What did you do?* |  |
| **7.** **Context** | Contextual elements considered important at the outset of introducing the intervention(s) | 4 |
| **8.** **Intervention(s)** | a. Description of the intervention(s) in sufficient detail that others could reproduce it b. Specifics of the team involved in the work | 4-9 |
| **9. Study of the Intervention(s)** | a. Approach chosen for assessing the impact of the intervention(s) b. Approach used to establish whether the observed outcomes were due to the intervention(s) | 10 |
| **10. Measures** | a. Measures chosen for studying processes and outcomes of the intervention(s), including rationale for choosing them, their operational definitions, and their validity and reliability b. Description of the approach to the ongoing assessment of contextual elements that contributed to the success, failure, efficiency, and cost c. Methods employed for assessing completeness and accuracy of data | 10 |
| **11. Analysis** | a. Qualitative and quantitative methods used to draw inferences from the data b. Methods for understanding variation within the data, including the   effects of time as a variable | 10 |
| **12. Ethical**  **Considerations** | Ethical aspects of implementing and studying the intervention(s) and how they were addressed, including, but not limited to, formal ethics review and potential conflict(s) of interest | NA |
| **Results** | *What did you find?* |  |
| **13. Results** | a. Initial steps of the intervention(s) and their evolution over time (*e.g.*, time-line diagram, flow chart, or table), including modifications made to the intervention during the project b. Details of the process measures and outcome c. Contextual elements that interacted with the intervention(s) d. Observed associations between outcomes, interventions, and relevant contextual elements e. Unintended consequences such as unexpected benefits, problems, failures, or costs associated with the intervention(s). f. Details about missing data | 10-12 |
| **Discussion** | *What does it mean?* |  |
| **14. Summary** | a. Key findings, including relevance to the rationale and specific aims b. Particular strengths of the project | 12 |

| **15. Interpretation** | a. Nature of the association between the intervention(s) and the outcomes b. Comparison of results with findings from other publications c. Impact of the project on people and systems d. Reasons for any differences between observed and anticipated outcomes, including the influence of context e. Costs and strategic trade-offs, including opportunity costs | 13-14 |
| --- | --- | --- |
| **16. Limitations** | a. Limits to the generalizability of the work b. Factors that might have limited internal validity such as confounding, bias, or imprecision in the design, methods, measurement, or analysis c. Efforts made to minimize and adjust for limitations | 14 |
| **17. Conclusions** | a. Usefulness of the work b. Sustainability c. Potential for spread to other contexts d. Implications for practice and for further study in the field e. Suggested next steps | 15 |
| **Other information** |  |  |
| **18. Funding** | Sources of funding that supported this work. Role, if any, of the funding organization in the design, implementation, interpretation, and reporting | 1 |

**Table 2. Glossary of key terms used in SQUIRE 2.0. This Glossary provides the intended meaning of selected words and phrases as they are used in the SQUIRE 2.0 Guidelines. They may, and often do, have different meanings in other disciplines, situations, and settings .**

**Assumptions**

Reasons for choosing the activities and tools used to bring about changes in healthcare services at the system level.

# Context

Physical and sociocultural makeup of the local environment (for example, external environmental factors, organizational dynamics, collaboration, resources, leadership, and the like), and the interpretation of these factors (“sense-making”) by the healthcare delivery professionals, patients, and caregivers that can affect the effectiveness and generalizability of intervention(s).

# Ethical aspects

The value of system-level initiatives relative to their potential for harm, burden, and cost to the stakeholders. Potential harms particularly associated with efforts to improve the quality, safety, and value of healthcare services include opportunity costs, invasion of privacy, and staff distress resulting from disclosure of poor performance.

# Generalizability

The likelihood that the intervention(s) in a particular report would produce similar results in other settings, situations, or environments (also referred to as external validity).

# Healthcare improvement

Any systematic effort intended to raise the quality, safety, and value of healthcare services, usually done at the system level. We encourage the use of this phrase rather than “quality improvement,” which often refers to more narrowly defined approaches.

# Inferences

The meaning of findings or data, as interpreted by the stakeholders in healthcare services – improvers, healthcare delivery professionals, and/or patients and families

# Initiative

A broad term that can refer to organization-wide programs, narrowly focused projects, or the details of specific interventions (for example, planning, execution, and assessment)

# Internal validity

Demonstrable, credible evidence for efficacy (meaningful impact or change) resulting from introduction of a specific intervention into a particular healthcare system.

# Intervention(s)

The specific activities and tools introduced into a healthcare system with the aim of changing its performance for the better. Complete description of an intervention includes its inputs, internal activities, and outputs (in the form of a logic model, for example), and the mechanism(s) by which these components are expected to produce changes in a system’s performance.

# Opportunity costs

Loss of the ability to perform other tasks or meet other responsibilities resulting from the diversion of resources needed to introduce, test, or sustain a particular improvement initiative

# Problem

Meaningful disruption, failure, inadequacy, distress, confusion or other dysfunction in a healthcare service delivery system that adversely affects patients, staff, or the system as a whole, or that prevents care from reaching its full potential

# Process

The routines and other activities through which healthcare services are delivered

# Rationale

Explanation of why particular intervention(s) were chosen and why it was expected to work, be sustainable, and be replicable elsewhere.

# Systems

The interrelated structures, people, processes, and activities that together create healthcare services for and with individual patients and populations. For example, systems exist from the personal self- care system of a patient, to the individual provider-patient dyad system, to the microsystem, to the macrosystem, and all the way to the market/social/insurance system. These levels are nested within each other.

# Theory or theories

Any “reason-giving” account that asserts causal relationships between variables (causal theory) or that makes sense of an otherwise obscure process or situation (explanatory theory). Theories come in many forms, and serve different purposes in the phases of improvement work. It is important to be explicit and well-founded about any informal and formal theory (or theories) that are used.
